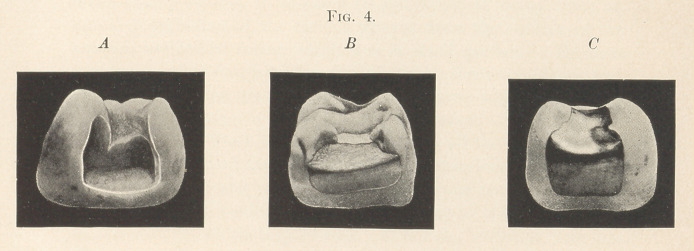# The Technique of Approximal Restorations with Gold in Posterior Teeth

**Published:** 1903-09

**Authors:** M. L. Rhein

**Affiliations:** New York City


### THE TECHNIQUE OF APPROXIMAL RESTORATIONS WITH GOLD IN POSTERIOR TEETH.[1]

BY M. L. RHEIN, M.D., D.D.S., NEW YORK CITY.

PROFESSOR BLACK's articles on operative dentistry, which have appeared at intervals during the last twelve years, have been the theme of many heated dental discussions. The scientific methods of cavity preparation which he has given us and his bold proclamation,

[1] Read before the Academy of Stomatology, Philadelphia, February 24, 1903.

"Extension for prevention," have acted as a firebrand thrown into a lot of dry timber. The member of the profession whose life is spent day by day, hour by hour, in untiring efforts to preserve man's dental organs, can never fail to find this subject interesting and inspiring.

"Extension for prevention" is no new doctrine to the older members of the profession in this city. In the short period of life in which Marshall H. Webb honored the city of Philadelphia by the practice of his profession he proclaimed this dogma far and wide. His words, spoken and treasured in print, attest this but feebly, compared to the herculean clinical instruction for which he was so famous. Much of his work remains to-day, a living testimony not only of his great skill, but of his genius in understanding where extension is or is not called for.

It may be as well to say, at the outset, that this is not intended as a plea for indiscriminate extension of cavity lines. On the contrary, it is freely admitted that no question which the operator has to meet is more difficult of correct solution than when and how freely extension should be practised. Its abuse is not our subject; but the wide-spread teaching of the doctrine has brought attention to the details of cavity preparation and gold packing.

For some years past the profession has been menaced with the great danger that the improper use of crowns and gold caps would in time make gold contour operations a lost art. This has been averted by the steadily increasing number of believers in the extension creed.

A careful investigation of methods used at clinics in inserting gold fillings will show to us some marked improvements in cavity preparation during this era. A number of faults have been detected and changes made which have placed cavity preparation on a more scientific basis. This good work has been largely fostered by the disciples of extension.

Whether the lines of the cavity are to be appreciably extended or not, has no bearing on the great advantages that are to be gained by discarding what has been found to be faulty, and adopting scientific methods of preparation. There was a time when retaining pits at the cervical borders were abandoned and diminutive holes made which were called starting-points. This lesser evil has passed with the greater, and the starting-points are known no more.

One of the most important changes in preparation has been the removal of the naturally rounded outlines of the cervical margin, especially at the angles. By means of either fissure or inverted cone burs the cervical wall is made perfectly flat, in order to form a stable foundation for the filling. This leaves a flat surface for the filling to rest upon, and as the operator deviates from this ideal the ability of the filling to withstand the strain of usage will be lessened. The buccal and lingual walls should start at right angles from the flattened surface and run in parallel lines from the flattened cervical seat to the crown of the tooth. Only in small fillings should these bucco-lingual walls be grooved. It must be remembered that all grooves or undercuts in these positions tend to weaken the supporting walls. The sides of these walls should remain, with the exception of the exterior bevelled margin and the interior dovetailed shape, as nearly straight as possible. The real anchorage seat of all fillings of this nature depends on the dovetailed occlusal step cut at right angles into the occlusal surface. (See Fig. 1.) This step, if cut into a non-carious occlusal surface, should be absolutely flat and not be made much thicker than the enamel itself, in order to preserve the strength of the walls. Frequently, however, the crown of the tooth has deep-seated caries as well.

In such cases it is best to fill this cavity separately, only up to the point where the right-angle step would naturally be. Then the approximate filling, when it reaches the proper height, will be packed and united against this flat floor of gold. In all such cases, where the walls are materially weakened, it is essential to guard against the breaking of the tooth or one of the walls. This is accomplished by cutting away a sufficient amount of these walls, so that they will be covered by gold of sufficient thickness to stand the wear of continued usage. A very important part of the operation is the polished bevelling of the enamel margin from the cervical border to the occlusal portion of the tooth; wherever it is feasible this bevel should extend around the outer periphery of all enamel margin. (See Fig. 2.)

Such work is well done by the gem cavity stones, finishing with sand-paper disks. The cavity itself may be prepared in an ideal manner, and yet recurrence of decay commence along the margins, if proper attention is not given to the bevelling of the enamel-rods. (See Fig. 2.) This bevelled surface should vary in size and shape, according to the strength of the walls. All these remarks about

With slight modification from Webb.

FIG. 1.

FIG. 2.

FIG. 3.

FIG. 4.

*A*

*B*

*C*

cavity preparation follow out the laws laid down by Professor Black, and for further detailed information on the subject, as to double steps, stress, etc., the reader is referred to his well-known articles on the subject.

Bearing in mind that all such cavities, after being properly prepared, are left in condition for the easiest introduction of gold, the following question is raised: Has equal progress been made in the methods of impacting the gold in cavities? The careful investigator can only reply in the negative to this inquiry. Method after method has been evolved, manifold forms of gold have been introduced, all done with the intention of enabling the operator to pack gold in a cavity with less expenditure of time and labor. Too frequently this saving in time and labor has been accomplished at the expense of the life of the filling, due to its lack of homogeneous compactness.

It is confidently asserted that gold fillings, at the present day, are not placed in teeth in what may be termed an ideal manner by as great a proportion of operators as in the time of Webb. While it is freely admitted that the most difficult portion of making a gold contour consists in properly shaping and preparing the cavity, it must not be forgotten that the insertion of the gold requires a considerable amount of time and patience and some little skill.

Professor Black, in one of his most recent articles on this subject, devotes considerable space in attributing the recurrence of caries to two causes,—"First, those due to the faulty manipulation of gold in packing, or the failure to make fillings completely water-tight; second, those cases of recurrence of decay from an actual rebeginning of the carious process upon the surface of the enamel, beside the filling which was in itself perfect." He then proceeds to state how to differentiate between these two causes, and almost takes it for granted that the first cause—leaky fillings due to faulty manipulation—is of minor importance. In fact, all the members of the Black creed write as though all the recurrences of decay that are found are due to insufficient extension.

If all recurrences of caries came under our notice as soon as they occurred, it would be comparatively easy to make a diagnosis of the class to which they belong. As a rule, sufficient time has elapsed to make such differentiation impossible.

It is just as permissible to make the following dogmatic statements. Faulty manipulation is most apt to occur nearest the cervical border. Such faults may be due to various forms of defective manipulation, but in such cases the commencement of the recurrence of caries may be said to begin within twenty-four hours. The slow development of such recurrence will depend on the state of immunity to caries existing at the time. Where comparative immune conditions exist, caries is liable to proceed under the filling in the gingival third, without being detected by average dental examination. When, finally, it manifests itself in no unmistaken form beyond the lines of the original cavity, who dares to state dogmatically that the caries has proceeded from recurrence on adjacent surface to the original cavity or *vice versa,* starting from a defective filling to the undermining and breaking down of the surface adjacent to the filling?

One of the most unfortunate features in the practice of Professor Black's disciples is this: Most of them appear to have adopted methods of inserting gold which tend too readily to produce defective stoppings. In their efforts to save time they have sacrificed the inherent strength of the plug. The old axiom holds as true here as elsewhere. A gold filling is no stronger than its weakest point. The very essence of Webb's success lay in his recognition of this law. What he said so often in society meetings on this subject are as true now as then, and they were true then. Quoting from the pages he wrote when he knew he was dying, he said, " Gold in the form of cylinders or pellets, and when in a non-cohesive condition, may be used so as to prevent decay in some so-called simple cases; but when really fine or first-class operations are to be performed, foil ought to be so carefuly prepared, introduced, and solidified that the operator can be positive that each piece has been firmly anchored in place or has adhered to that already in position, and, being certain of this, the whole filling can be made solid and uniform in density, and the organ operated upon fully restored to usefulness."

There is no intention of denying the fact that simple cavities can be sealed and preserved by the use of non-cohesive gold used alone and in combination with cohesive gold. Where, however, the greatest resistance possible is demanded against the one hundred to three hundred pounds of pressure used in mastication, it is reasonable to suppose that the more solid the stopping, the greater its strength and durability.

In contour restorations of the posterior teeth, the greatest resisting power possible of attainment is always demanded. In writing on this subject, Professor Black, referring to the greater amount of strain a natural tooth will bear than any filling, says that "all that gold will bear is all that it will bear under a crushing strain. The weakest portion will give first, whether it be the cervical or occlusal, and either will destroy the filling." In another place he says, where a very heavy filling is to be supported, "platinum gold" packed in the occlusal step is, on account of its hardness, much more durable. If the extra hardness of "platinum gold" will give a better plane of resistance to the crushing strain of mastication, the same line of reasoning will hold good in the comparison of cohesive gold fillings with those made up of a combination of cohesive and non-cohesive foil.

The question resolves itself into the amount of crushing strain a filling will bear that is made up from beginning to end of pieces of cohesive gold-foil solidly packed together, so that the finished work is one solid homogeneous mass of gold, the molecules of which are as inseparable as would be found in an ingot of melted gold. Compare this with a filling composed of non-cohesive and cohesive foil held together by the mechanical locking of the various pieces of gold, which can be readily separated by means of sufficient pressure, and the superiority of the former filling in its ability to withstand the crushing strain of mastication is at once apparent.

Having determined, therefore, that the completed filling should be one solid plug, your attention is invited to one method of inserting the same. That recurrence of decay at the cervical border destroys the usefulness of most gold fillings has long been an admitted fact. It is here at the beginning of the filling the operator must exercise the greatest care. It is understood that a solid flat floor has been prepared free from any checks or imperfections of the enamel margins. The axial walls, though perpendicularly parallel, are slightly dovetailed towards the pulpal wall, so that as the filling advances in size it is more securely locked into position by the prepared shape of the cavity.

This locking embrasure of the cavity should be formed by a gradual sloping of the wall of the dentine from the enamel margin towards the pulpal wall. It should always be free from any narrow or deep undercuts, which not only weaken the walls, but tend materially to retard the speedy packing of the gold-foil. The cavity is now washed with a few drops of ten per cent. solution of formalin and then thoroughly dried. A very small piece of one of the plastic forms of gold, like "moss fibre" or "De Trey's" is placed on the electric gold annealer. A small amount of oxyphosphate of zinc having been thoroughly mixed to a thick creamy consistency, an amount generally equal to the size of the head of a pin is taken on the point of a broach and carefully placed along the inner half of the floor, care being taken to keep the margins entirely free from any cement. The small piece of annealed plastic gold is now carefully laid in position over the cement. With a small round burnisher (kept thoroughly polished) the gold is gently and evenly worked into the film of cement. It requires a little practice to be able to properly burnish this small starting-piece of gold into proper position.

The pressure on the gold must be extremely delicate, evenly divided, and always towards the pulpal wall. No cement should ever appear beyond the gold towards the enamel margins, nor on the upper surface of the gold, nor come into contact with the burnisher. The cavity is now ready for the strip of freshly annealed gold-foil a little narrower than the floor of the cavity. This is at once malleted against the gold starting-point, which is cemented fast to the dentinal floor. It is generally preferable to wait a few moments for the small amount of cement to harden, and this time can be utilized by cutting the gold of desired thicknesses, from No. 30 to No. 60, into strips of such width as can be most satisfactorily used.

A capable assistant employed to place the proper strips of gold on the electric annealer, and feed the same, will be found invaluable in saving time for the operator. The floor of the cavity is now covered evenly by gold-foil malleted and condensed solidly against the flat surface, care being taken that a piece of gold should always intervene between the plugger and the tooth-substance.

The filling is now brought in an even manner from the bottom upward, advancing no part of the gold beyond another part. This requisite evenness of surface is best accomplished by wiping the gold with the plugger, hammering away from side to side. To accomplish this in the most satisfactory manner, it is essential that the blows should be delivered in very rapid rotation, from two to four thousand per minute. There should be no unevenness of power in the blow and it should always be under the perfect control of the operator.

The ideal mallet for twenty-five years has been the electro-magnetic mallet invented by W. G. A. Bonwill and improved by Marshall H. Webb, and more recently by George L. Harrison. Webb, in speaking of the mallet, says, "The packing instrument should be touched upon or placed (not pressed) against the gold in a manner similar to that of making dots on paper with a pencil. Light, medium, or hard blows can be made without changing the adjustment of the instrument, as fine or heavy lines are made on paper with a pen. When the electro-magnetic mallet is operated and guided as here indicated, gold can be carried against and over the margins (even frail edges) of enamel without fracturing them." In speaking of its superiority, he says, "This is true of the work of the electro-magnetic mallet, because, to expel the air from between the particles of foil and place them in absolute contact in every given piece or body of gold, a certain number of blows of given force are necessary; and to thus go over the whole of each piece being impacted by any other known method would require the expenditure of more time and greater effort. That gold be made compact it is not so desirable that a heavy blow simply be struck as it is necessary that rapid, regular, and only moderately heavy blows be skilfully given to each piece of foil. By no other method can this be done so well and so perfectly as with the electro-magnetic mallet."

These words remain as true to-day as when written by that gifted operator over twenty years ago. Every one who knew Webb realized how thoroughly he believed in the electro-magnetic mallet, so much so that it seemed a part of him. It might almost be said that he died in the cause of propagating the benefits that would ensue from the use of this instrument.

In his time it was necessary to depend on crude forms of inefficient batteries for power which interfered materially with the general adoption of the mallet. The adjustments of the instrument were easily disturbed, and required a thorough comprehension of its construction to readjust.

Recently, Mr. Harrison, of the S. S. White Company, has succeeded in improving the mallet to such an extent that all the former objections to the instrument's getting out of order and not working properly have been removed. The improvements in batteries and the ability to do without their use entirely has removed the most cogent opposition to the adoption of this instrument.

That the improved electro-magnetic mallet is not better known to-day is due to the fact that we have no Webb to cry its praises from the Atlantic to the Pacific. It is generally very unsatisfactory to attempt to change methods that have produced good work for a long number of years. To the young men, however, who are striving to produce gold restorations that will be time enduring, the instrument is recommended as possessing qualifications superior to that of any other form. Notwithstanding the disadvantages attending the use of the mallet twenty years ago, there are many of our best operators to whom it has always been their chief mainstay.

I wrote to three exceptionally good operators, who have used the mallet for over twenty-two years, and their replies are as enthusiastic in its favor to-day as they have always been. It affords me great pleasure to read what Dr. R. H. Hofheinz, President of the New York State Dental Society, has written to me on this subject:

"The question regarding the electro-magnetic mallet must be considered from two stand-points,—first, its advantages; second, its disadvantages. Among the disadvantages I should count, first, the necessity of an assistant. I cannot imagine myself using this instrument without the help of a skilled assistant; second, strictly cohesive gold in all cavities from beginning to the end of the operation; third, its restricted use to fair and large sized and accessible cavities; fourth, the disagreeable noise to some patients.

"The advantages are, first, rapidity of work; second, uniform condensation of the gold; third, the secure condensation of gold against the margins of the cavity such as no other condensing methods afford; fourth, the benign response from a tender peridental membrane. When all other condensing methods, including hand-pressure, produce pain upon the peridental membrane (whatever may have been the cause of its nervous hypersusceptibility), the stroke of the electric mallet will be borne by that vascular tissue without pain; fifth, there are some people to whom the continuous noise is far preferable to that of other mallets; sixth, I may add that the frailest teeth are never filled with more safety than by the use of the electric mallet; seventh, no mallet is less apt to produce cleavage of the enamel."

Dr. W. C. Wendel and Dr. Charles Southwell, of Milwaukee, two of my classmates, have written with the same amount of enthusiasm. Dr. Southwell, among other things, writes: "The patient in the chair and the tooth in the socket cannot be disturbed by any method that deals gently with frail walls. The introduction of the hand-mallet and automatic mallet as prime methods by the colleges is a source of untold and unnecessary mischief. They should be taught as secondary methods only, useful in the event of a break in the equipment of the prime methods."

With the aid of the electro-magnetic mallet it is possible to make the largest of restorations without unnecessarily tiring the patient. The sittings can be made as short as may be necessary for the well-being of any individual. On account of its perfection of condensing properties the operation can be stopped at any point and gutta-percha inserted temporarily. Upon the return of the patient after the rubber dam has been applied, the superficial layer of gold has only to be burred away, and freshly annealed foil can be at once added with fully as perfect cohesion as if the filling had just been commenced. The ability to do this is the one great test of the proper condensation of gold by any form of instrument. In fact, the best results are generally attained where the lower third or half of the filling is inserted at one sitting and the operation completed at a later time.

In the majority of contour restorations the surfaces of both teeth are involved. In such cases it is most advantageous to fill the gingival third of both teeth at the same sitting. After the filling has been well started a matrix may often be used to advantage. This should serve merely as a convenience in shaping and in no wise be depended upon as a support to the filling. On this account, wherever a matrix is used it should be occasionally removed and the filling carefully tested in order to determine that it is held properly in place by its own walls and on its own foundation. It is an important essential of a satisfactory contour that all of the exposed surface of the gold should be so carefully finished as to resemble polished enamel. To no portion of the filling is this ideal finish more important than at the cervical margin. Care must be taken that all overhanging bits of gold should be removed and the polishing done without in any way defacing or marring the enamel margins.

It is questionable whether the proper finishing of this gingival third is not a more influential prophylactic than excessive extension would be without such a perfect finish. Consequently it must be conceded that this ideal finishing of gold is demanded if the filling is expected to be a permanent one. When the filling has been completed with its proper contact points, it is difficult and sometimes impossible to do this in the manner described. Consequently the polishing of the gingival third or half should be done as soon as the filling has been built up to the point where the convex slope of contour has its beginning. (See Fig 3.) Done at this time, the operator has all the advantage of plenty of room and space, and is not worried by any fear of destroying the contour shape.

It is generally impossible to do this finishing thoroughly without serious injury to the rubber dam, and the day's work may just as well stop at this point. Pink base-plate gutta-percha carefully packed in the remaining portion of the cavities will tend towards giving a better separation at the next sitting. Then, after the rubber dam has been applied, a fixed wedge of some character is used at the cervix in order to maintain sufficient separation. With a sharp bur the last film of gold-foil is removed and freshly annealed gold-foil is at once malleted successfully against the freshened gold surface. If the filling is large enough to demand it, the operation may in this manner be divided into any number of sittings and still become at the end one solid homogeneous plug of gold inseparable at any point.' This ability to divide the insertion of the gold into any number of short sittings appears not to be generally understood. It is a great boon to many patients who earnestly desire the most stable operation but who dread lengthy sittings. The remainder of the filling is now built up in the same even manner, carefully watching the reproduction in gold of the convex contact points.

Matrices can be used here to good advantage. They should be removed frequently and replaced with thinner ones, until the very thinnest is used at the place mapped out for the marble-like contact points. These contact points can be made natural in most cases. There are, however, some cases of irregular teeth when the interproximal space is exaggerated, and here the ideal contact points must be abandoned to such shaped contact points as each irregular case may demand.

When the packing of the gold has been completed, the teeth should be slightly wedged apart, and the contoured portion of the filling finished with the same care as was given to the finishing of the gingival portion. (See Fig. 4.) For this purpose fine chisels and files, supplemented by sand-paper strips and disks, are most efficacious. The final polishing can then be given with very fine cuttle-fish strips and disks which have been covered by a thin coating of vaseline. The occlusal portion of the filling is best finished by means of properly shaped plug-finishing burs, which by being kept wet are often sufficient to give the final finished polished surface. If not, this can be supplemented by fine pumice and wooden or moosehide points. The separator is now removed, the teeth come tightly together at their golden contact points, and there stands revealed the polished golden counterpart of the lost tooth-substance.

NOTE.—The sketch of upper teeth is taken from Webb's book, and distinctly illustrates how thoroughly he believed in extension. The sketch has been slightly modified in the way of removing the cervico-buccal and cervico-lingual curve and replacing same with straight walls.

## Figures and Tables

**Figure f1:**
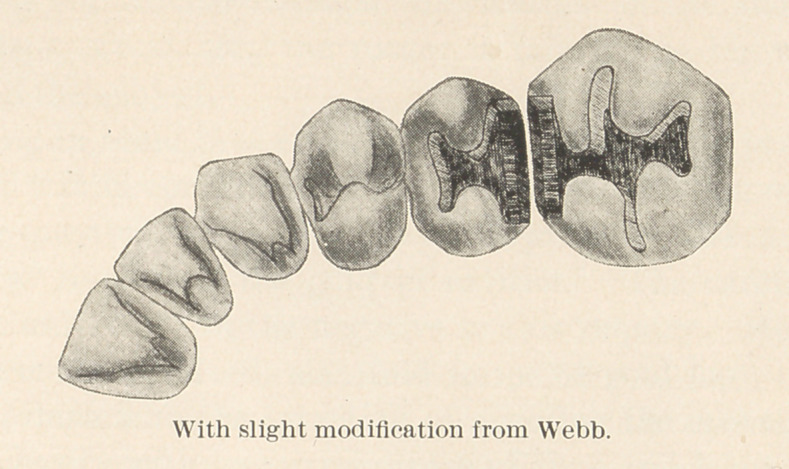


**Fig. 1. f2:**
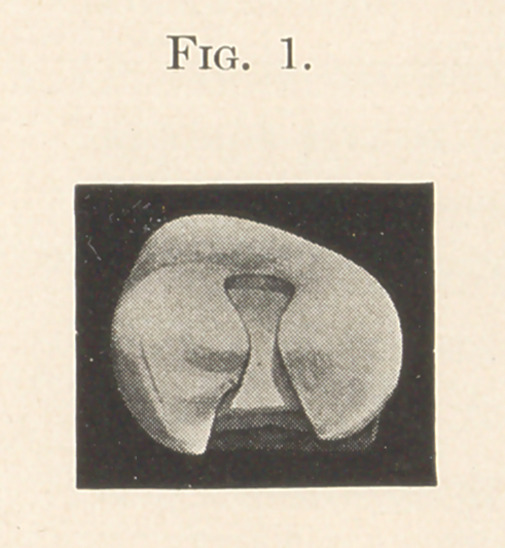


**Fig. 2. f3:**
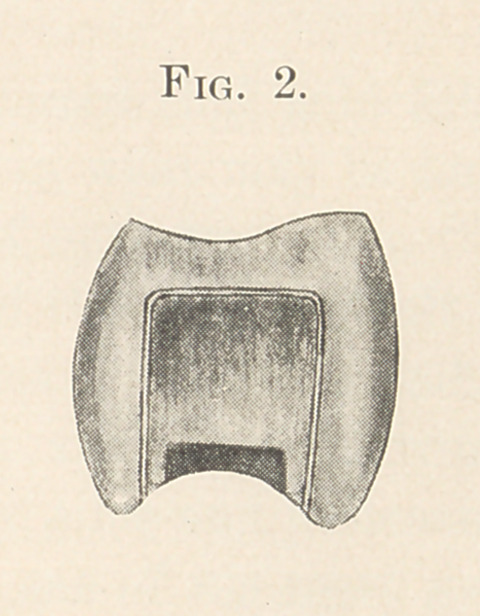


**Fig. 3. f4:**
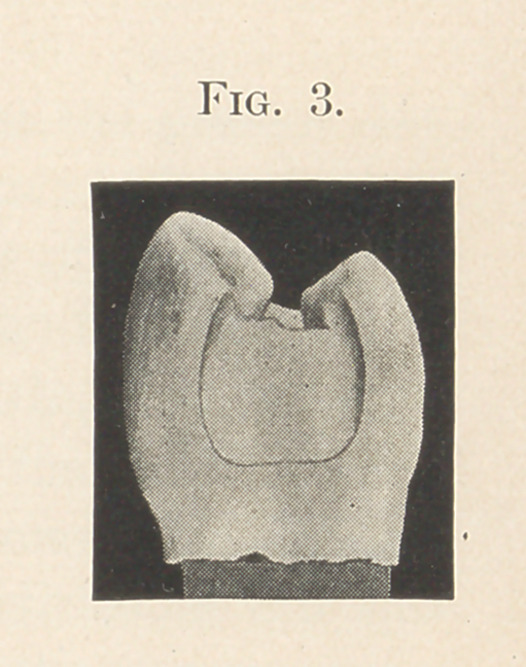


**Fig. 4. f5:**